# First case of Dolutegravir and Darunavir/r multi drug-resistant HIV-1 in Cameroon following exposure to Raltegravir: lessons and implications in the era of transition to Dolutegravir-based regimens

**DOI:** 10.1186/s13756-020-00799-2

**Published:** 2020-08-26

**Authors:** Joseph Fokam, Desire Takou, Ezechiel Ngoufack Jagni Semengue, Georges Teto, Grace Beloumou, Beatrice Dambaya, Maria-Mercedes Santoro, Leonella Mossiang, Serge Clotaire Billong, Fatim Cham, Samuel Martin Sosso, Edith Saounde Temgoua, Aubin Joseph Nanfack, Sylvie Moudourou, Nelly Kamgaing, Rachel Kamgaing, Joelle Nounouce Ngako Pamen, Mireille Mpoudi Ngole Etame, Anne-Cecile Z.-K. Bissek, Jean-Bosco N. Elat, Emmanuel Eben Moussi, Vittorio Colizzi, Carlo-Federico Perno, Alexis Ndjolo, On behalf of the VIROFORUM

**Affiliations:** 1 Chantal BIYA International Reference Centre for research on HIV/AIDS prevention and management, Yaoundé, Cameroon; 2 Faculty of Health Sciences, University of Buea, Buea, Cameroon; 3 National HIV Drug Resistance Working Group, Ministry of Public Health, Yaoundé, Cameroon; 4 Faculty of Medicine and Biomedical Sciences, University of Yaoundé I, Yaoundé, Cameroon; 5 University of Rome Tor Vergata, Rome, Italy; 6 Evangelic University of Cameroon, Bandjoun, Cameroon; 7 HIV Treatment Centre, Yaoundé Central Hospital, Yaoundé, Cameroon; 8 Central Technical Group, National AIDS Control Committee, Yaoundé, Cameroon; 9 World Health Organisation, Regional Office for Africa (AFRO), Brazzaville, Congo; 10 Department of Disease, Epidemics and Pandemics Control, Ministry of Public Health, Yaoundé, Cameroon; 11 HIV Treatment Centre, Military Hospital, Yaoundé, Cameroon; 12 Division of Health Operational Research, Yaoundé, Cameroon; 13 University of Milan, Milan, Italy

**Keywords:** HIV drug resistance, Integrase inhibitors, Protease inhibitors, Viral tropism, Cameroon

## Abstract

**Background:**

Sub-Saharan African countries are transitioning to dolutegravir-based regimens, even for patients with extensive previous drug exposure, including first-generation integrase strand-transfer inhibitors (INSTI) such as raltegravir. Such exposure might have implications on cross-resistance to dolutegravir-based antiretroviral therapies (ART).

**Case presentation:**

We report a 65 years old Cameroonian, previously exposed to raltegravir, and failing on third-line treatment with multi-drug resistance to darunavir/r and dolutegravir. Genotypic resistance testing (GRT) and viral tropism were performed during monitoring time points. The patient initiated ART in August 2007. At the time point of the first (29.04.2010), second (01.12.2017) and third (08.08.2019) GRT, prior ART exposure included 3TC, d4T, NVP and EFV; additionally TDF, DRV/r and RAL; and additionally ABC and DTG respectively. First GRT revealed mutations associated with resistance only to first-generation Non-nucleoside reverse transcriptase inhibitors (NNRTI). Second GRT revealed mutations associated with high-level resistance to all NRTIs, first generation NNRTIs, all ritonavir boosted protease inhibitors (PI/r), and all INSTI, while viral tropism (using geno2pheno) revealed a CCR5-tropic virus with a false positive rate (FPR) of 60.9% suggesting effectiveness of maraviroc (MRV). The third GRT showed high-level resistance to NRTI, NNRTI, all PI and all INSTI, with additional mutations (H221HY for NNRTI and S147G for INSTI), and a CCR5-tropic virus with a slightly reduced FPR (57.0%). Without any locally available active therapeutic option, the patient has been on a maintenance therapy with “DRV/r (600mg x 2/day)+TDF+3TC” and patient/family-centered adherence has been reinforced. Since the first viral load (VL) measurement in 2010, the patient has had 12 VL tests with the VL ranging from 4.97 Log to 6.44 Log copies/mL and the CD4 count never exceeded 200 cells/μL.

**Conclusions:**

As African countries transition to dolutegravir-based regimens, prior raltegravir-exposure may prompt selection (and potential transmission) of dolutegravir-resistance, supporting case surveillance.

## Background

Management of treatment-experienced HIV-patients remains very challenging in resource-limited settings where drug options are limited, poor adherence is frequent, and treatment monitoring (viral load essentially) remains sub-optimal [1, 2]. ART program was launched in Cameroon by May 2003, and genotyping drug resistance testing (GRT) is recommended essentially after failing a PI/r-based regimen [3]. Following the recent World Health Organisation (WHO) recommendations, most resource-limited settings, including Cameroon, are transitioning to dolutegravir (DTG)-based regimens for first-, second- and/or third-line ART [3], with no evidence of DTG-resistance reported in West-Central Africa [1, 3], and fewer cases in East Africa [4]. Of note, few studies reported lower rates of cross-resistance between raltegravir (first-generation integrase strand-transfer inhibitor) and dolutegravir (second-generation integrase strand transfer inhibitor) in Europe [5–8]. Exposure to raltegravir has been previously reported in managing patients with multi-resistant HIV in Africa, but little is known on their effects after transitioning to dolutegarvir-containing regimens [9]. Understanding the implications of prior exposure to raltegravir in routine clinical practice would serve as footprint for optimal ART-policies in this transition era, particularly in West and Central Africa and also in all resource-limited settings like Cameroon.

## Case presentation

**Fig. 1:**
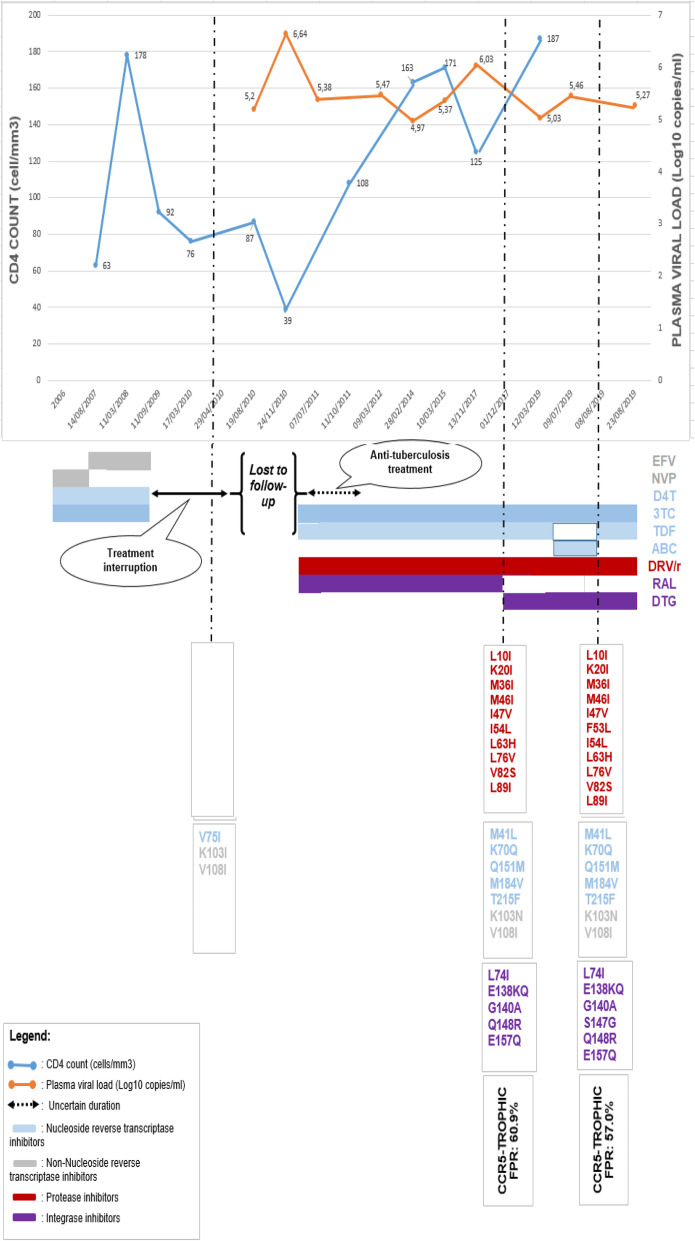
CD4-cell and viral load monitoring, ART regimens, and resistance profiles since ART-initiation

This is a 65 years old married man, infected with HIV-1 CRF18_cpx. Therapeutic history, VL, CD4 cell counts and genotypic profiles are summarised in Fig. 1.

The patient was diagnosed with HIV in 2002 and has been on ART for 14 years (since 2006). His initial ART regimen was stavudine (d4T) + lamivudine (3TC) + nevirapine (NVP) from August 2006 to August 2007. NVP was subsequently replaced by efavirenz (EFV) from August 2007 to August 2009. From August 2009, the patient voluntarily stopped treatment and returned to the clinic in 2010 for consultation. In 2010, the patient had a high VL (157,290 RNA copies/ml) and very-low CD4 cell count (87 cells/mm^3^). Prior to ART re-initiation in 2010, genotypic resistance testing (GRT) results, interpreted throughout using Stanford HIVDB (http://www.hivdb.stanford.edu) and International AIDS Society mutations list, showed the presence of one resistance associated mutation (RAM) to NRTI (V75I), two non-NRTI RAMs (K103I, V108I). With this mutational profile, the patient was recommended PI/r + 2NRTI but we have no precision on antiretrovirals used thereafter. From 2011 to 2012, the patient was diagnosed co-infected with pulmonary tuberculosis and treated accordingly, classified as WHO clinical stage 3. After completing anti-tuberculosis treatment in 2012, he was lost to follow-up (duration unclear) and later returned for clinical consultation. Following clinical assessment, he received a regimen consisting of super-boosted ritonavir with darunavir (DRV/r) + raltegravir (RAL) + tenofovir (TDF) +3TC (unclear start date) till November 2017, a period during which adherence was suboptimal (assessed by self-reporting). While completing anti-tuberculosis treatment the patient discontinued HIV-treatment. Following clinical assessment, he was re-initiated with a regimen consisting of ritonavir boosted darunavir (DRV/r) + raltegravir (RAL) + tenofovir (TDF) +3TC (unclear start date) till November 2017; a period during which adherence was suboptimal. A second GRT was performed and the resulting mutational profile suggested DRV/r(600 mg × 2/day) + DTG(50 mg × 2/day), possibly associated with ritonavir-boosted atazanavir (ATV/r) for its potential capacity to enhance DTG concentrations (https://www.hiv-druginteractions.org/drug_queries/326049/drug_query_interactions) as an optimal regimen. However, the patient was prescribed DRV/r(600 mg × 2/day) + DTG(50 mg × 1/day) + TDF + 3TC from November 2017 to May 2019. In May 2019, the patient was empirically switched to DRV/r(600 mg × 2/day) + DTG(50 mg × 1/day) + abacavir (ABC) +3TC. Then, ABC was changed to TDF until August 2019 when the third GRT was performed.

Overall, reported VLs remained around 5 Log_10_ copies/mL while CD4-cell counts were consistently < 200 cells/mm^3^. Adherence to clinic appointments and drug refill was inconsistent, in spite of adherence supports from health care providers. Apart from anorexia and a 2.8% weight-loss reported over an undefined period, a physical examination, performed in May 2019, was unremarkable [3].

Following GRTs performed on HIV-1 “*pol”* gene for detecting RAMs and gp120 V3-loop for coreceptor-usage (using the geno2pheno method [https://coreceptor.geno2pheno.org/]) at three different time points [10, 11], phylogeny consistently revealed the virus to be HIV-1 CRF18_cpx. All sequences generated were submitted to GeneBank (MN52015 – MN520219).

The first GRT (April 29, 2010) covered the protease (PR) and reverse transcriptase (RT) regions, with high-level resistance to first-generation NNRTI (K103I, V108I) and no major-RAM to NRTI and PI/r.

The second GRT (December 01, 2017) covered PR and RT regions. Detected RAMs were M41L, K70Q, V75I, Q151M, M184V and T215F for NRTI; K103N and V108I for NNRTI; and L10F, K20I, M36I, M46I, I47V, I54L, L63H, L76V, V82S and L89I for PI/r. These mutations revealed high-level resistance to all NRTIs, first generation NNRTIs (EFV and NVP) and to all PI/r, including DRV/r. Another GRT, covering HIV-1 integrase (IN) region, performed on the sample plasma aliquot, revealed major RAMs, L74I, E138KQ, G140A, Q148R, and E157Q, to integrase strand transfer inhibitors (INSTI) including raltegravir, dolutegravir, bictegravir and elvitegravir. Viral tropism revealed a CCR5-tropic virus, defined by a false positive rate (FPR) of 60.9%, suggesting eligibility for maraviroc (MRV).

The third GRT (August 08, 2019), encompassing PR, RT and IN regions, showed high-level resistance to NRTI, NNRTI, PI and all INSTI. Additional RAMs were H221HY for NNRTI and S147G for INSTI. Viral tropism was also re-performed and demonstrated CCR5-tropic virus with a slightly reduced FPR (57.0%). Pending access to required innovative regimen, a salvage therapy guided by GRT was recommended, consisting of either “DRV/r(600mg x 2/day), saquinavir/r, TDF+3TC”, or “3TC-monotherapy” which maintains M184V and as such limits the viral replicative fitness. None of the recommendations; in November 2019, VL appeared relatively stable (5.54 Log copies/mL) under “DRV/r(600mg x 2/day)+TDF+3TC” and with an active family-centered adherence monitoring. Following intensified/close adherence strategy implemented, the patient is compliant to treatment but the latest plasma viral load remains high, with a slight increment (5.86 Log copies/mL on January 27, 2020), which confirms the need of innovative drugs and close case surveillance, including MRV that is not locally available [3].

## Discussion

To the best of our knowledge, this is the very first clinical case in West and Central Africa of complete four-class multidrug resistance to all antiretrovirals available in resource-limited settings with complete loss of DRV/r and DTG efficacies. Of note, previous report from Cameroon showed a case of successful raltegravir-containing ART for managing a case of multidrug-class-resistance [9]. Thus, patients with similar therapeutic management within the country might merit a close monitoring to ensure a successful management in case of ART failure with multi-drug resistance [9]. Fewer cases of four-class HIVDR have been recently reported mainly in East and southern African countries, with previous RAL-exposure contributing to ineffectiveness of DTG [4, 12–14]. Though these previously reported clinical cases were attributed to multi-factorial events, the present clinical case was due to recurrent poor adherence, leading to selection and accumulation of RAMs, followed by exposure to suboptimal drug regimens [14–16]. Specifically, previous exposure to RAL (a drug with a low-genetic barrier to resistance) in a context of poor adherence contributed to selecting mutations (E138KQ, G140A, Q148R) conferring cross-resistance to DTG [4, 15, 16]. In a context with 60–80% ADR after ART failure and with documented to RAL has been documented, limiting the use of RAL would preserve DTG for long-term use [1, 16]; and in case of previous exposure to RAL, transition to DTG-based regimens should be guided by GRT in people without VL-suppression [3] and if adopted, DTG-dose should be appropriate [6, 7]. In addition to non-adherence, consistent low CD4-cell counts (despite a relative increase of CD4 after a nadir of 39cells/mm^3^ is attribuable to the use of more potent antiretrovirals), high-VL since ART-initiation, and advanced-age (i.e. > 60 years) are risk-factors that might have contributed to ART-failure [14–16]. Additionally, the recombinant virus (HIV-1 CRF18_cpx) may explain to a certain extend the rapid emergence of drug-resistance and the predictive CCR5-tropism [17]. The case finding calls essentially for surveillance of acquired (ADR) drug resistance to INSTI in the DTG-era [1–3]. As patient remains clinically stable and sexually active, special patient/family-centered adherence approaches are necessary to limit risk of transmission of four-class drug resistant virus, which may have implications for scale-up of DTG-based ART in resource-limited settings [1, 4, 12–15]. Without any active therapeutic option locally, use of innovative drug combinations consisting preferentially of enfuvirtide, MRV and ibalizumab, is highly recommended (https://www.hiv-druginteractions.org/drug_queries/326049/drug_query_interactions). Also, as patients on DTG-containing regimens with high baseline VL (> 500,000 copies/mL) might experience challenges in viral suppression, close monitoring as well as specific case surveillance should be required even in resource-limited settings like Cameroon [18].

## Conclusion

In the era of transition to DTG-based regimens in resource-limited settings, this clinical case highlights the need for routine ADR population-based surveys to understand the extent of HIVDR to INSTI. Importantly, optimised care/treatment on DTG-containing regimen, alongside adherence support and close VL monitoring, would sustain the efficacy of this therapeutic option. Finally, individual case surveillance after failure to third-line requires genotyping and policies for access to newer drugs in resource-limited settings with similar ART landscapes.

## Data Availability

Data and materials are fully available in the manuscript text and in the figure provided. Sequence data generated are deposited in the public repository of GeneBank, under the following accession numbers from MN52015 to MN520219.

## Abbreviations

3TC: Emtricitabine
ABC: Abacavir
ADR: Acquired drug resistance
AIDS: Acquired Immunodeficiency Syndrome
ART: Antiretroviral therapy
ATV/r: Ritonavir boosted Atazanavir
CCR5: C-C chemokine receptor type 5
CD4: Cluster of differentiation four
CRF18_cpx: Circulating recombinant form complex 18
D4T: Stavudine
DRV/r: Ritonavir boosted Darunavir
DTG: Dolutegravir
EFV: Efavirenz
FPR: False positive rate
GP 120: Envelope glycoprotein 120
GRT: Genotypic resistance testing
HIV: Human immunodeficiency virus
HIVDR: HIV drug resistance
IN: Integrase
INSTI: Integrase strand transfer inhibitor
LPV/r: Ritonavir boosted Lopinavir
MRV: Maraviroc
NNRTI: Non-nucleoside reverse-transcriptase inhibitor
NRTI: Nucleoside reverse-transcriptase inhibitor
NVP: Nevirapine
PDR: Pretreatment drug resistance
PI/r: ritonavir boosted protease inhibitor
Pol: Polymerase
PR: Protease
RAL: Raltegravir
RAMs: Resistance-associated mutations
RNA: Ribonucleic acid
RT: Reverse transcriptase
SSA: Sub-Saharan Africa
TDF: Tenofovir disoproxyl fumarate
V3: Envelope variable region 3
VL: Viral load
WHO: World Health Organization
